# How may cultural and political ideals cause moral distress in acute psychiatry? A qualitative study

**DOI:** 10.1186/s12888-022-03832-3

**Published:** 2022-03-23

**Authors:** Trine-Lise Jansen, Lars Johan Danbolt, Ingrid Hanssen, Marit Helene Hem

**Affiliations:** 1 Norway, MF Norwegian School of Theology, Religion and Society, Lovisenberg Diakonale Høgskole (Lovisenberg Diaconal University College), Oslo, Norway; 2 MF Norwegian School of Theology, Religion and Society, Oslo, Norway; 3 Lovisenberg Diakonale Høgskole (Lovisenberg Diaconal University College), Oslo, Norway; 4 VID Specialized University, Oslo, Norway

**Keywords:** Moral distress, Acute psychiatry, Coercion, Cultural ideals, Political ideals, Psychiatric nursing, Mental health nursing

## Abstract

**Background:**

There is growing public criticism of the use of restraints or coercion. Demands for strengthened patient participation and prevention of coercive measures in mental health care has become a priority for care professionals, researchers, and policymakers in Norway, as in many other countries. We have studied in what ways this current ideal of reducing the use of restraints or coercion and attempting to practice in a least restrictive manner may raise morals issues and create experiences of moral distress in nurses working in acute psychiatric contexts.

**Methods:**

Qualitative interview study, individual and focus group interviews, with altogether 30 nurses working in acute psychiatric wards in two mental health hospitals in Norway. Interviews were recorded and transcribed. A thematic analytic approach was chosen.

**Results:**

While nurses sense a strong expectation to minimise the use of restraints/coercion, patients on acute psychiatric wards are being increasingly ill with a greater tendency to violence. This creates moral doubt and dilemmas regarding how much nurses should endure on their own and their patients’ behalf and may expose patients and healthcare personnel to greater risk of violence. Nurses worry that new legislation and ideals may prevent acutely mentally ill and vulnerable patients from receiving the treatment they need as well as their ability to create a psychological safe climate on the ward. Furthermore, persuading the patient to stay on the ward can cause guilt and uneasiness. Inadequate resources function as external constraints that may frustrate nurses from realising the treatment ideals set before them.

**Conclusions:**

Mental health nurses working in acute psychiatric care are involved in a complex interplay between political and professional ideals to reduce the use of coercion while being responsible for the safety of both patients and staff as well as creating a therapeutic atmosphere. External constraints like inadequate resources may furthermore hinder the healthcare workers/nurses from realising the treatment ideals set before them. Caught in the middle nurses may experience moral distress that may lead to physical discomfort, uneasiness and feelings of guilt, shame, and defeat.

Pressure on nurses and care providers to reduce or eliminate the use of coercion and reduction of health care spending are incompatible demands.

## Background

Acute psychiatric units are specialised places for persons needing voluntary or involuntary short-term treatment during an *acute* phase of psychiatric illness. Admission to an acute in-patient unit depends on the severity of the psychiatric symptoms, the person’s level of distress and the risk of harm to self or others [1].

The development of legislation concerning the mentally ill are closely related to the development within the society at large. With every new legal reform, the treatment of the «mad», «insane», and «mentally ill» has, as these labels imply, become more humane. The European Council has ambitious goals regarding discontinuation of coercive measures within psychiatric healthcare in Europe. This follows the United Nation’s Convention on the Rights of Persons with Disabilities [2]. Concurrently there is growing public criticism of the use of restraints and coercion together with demands for strengthened patient participation, and scepticism toward medical treatment. Prevention of restraining or coercive measures has become a priority for care professionals, researchers and policymakers [3], and reduced use of seclusion is now widely identified as a quality issue for mental health services [4].

In Norway, restraining and coercive treatment measures are only to be used when unavoidable. In 2017 a new *Law on Patients’ Rights *[5] was passed which established that only patients incapable of giving competent consent may be involuntarily admitted for psychiatric treatment. This law’s aim is to strengthen psychiatric patients’ autonomy and legal rights. In 2019 the *Coercion Restriction Law* was presented for the Norwegian Parliament. According to this legislative proposal the use of mechanical restraints should be discontinued within three years [6].

Although well intentioned, the proposed legislation with its aim to reduce the use of restraints and coercion is unspecific regarding when coercion may be used. Added to this, diffuse political and therapeutic ideals that are difficult to realise may increase the vulnerability and moral distress experienced by nurses working in mental health wards [7].

The concept *moral distress* is attributed to Jameton [8] (1984) and may be defined as an unpleasant feeling or a psychological imbalance which arises when one knows what the ethical right action in a certain situation is, but internal or external restraining factors make it not possible to act accordingly. Political and/or therapeutic ideals may be among these restraining factors. In line with other researchers [9, 10] we find that in complex care settings it may be difficult to know what the morally right course of action is. In recent years there has been an increasing debate about the conceptualization of moral distress [11]. Based on our empirical findings we will argue for a broader definition of the concept in which contexts where caregivers face moral dilemmas or experience moral doubt are included. Moral doubt might be described as not knowing or being uncertain whether something is morally right or justified [12]. Research indicates that the nurses find themselves in moral doubt over time, experience moral distress [13].

Unresolved moral distress may lead to for instance feelings of guilt, bad conscience, sadness, powerlessness, emotional numbness, shame, cynicism, despondency, anger, angst, self-criticism, resignation and may violate one’s integrity [13–16]. Nurses who experience moral distress tend to withdraw emotionally from patients [15, 17] and disconnect from themselves and others [13, 14, 18]. Common related physical symptomatology are fatigue, exhaustion, headaches, stomach pain, sleeplessness, weight changes and palpitations [14, 19, 20]. Thus moral distress may cause staff turnover [17, 21, 22], burn-out [16, 20, 22, 23], and ultimately, is harmful to patients [15, 16, 22].

### Aim and research questions

The aim of this paper is to investigate whether diffuse political and therapeutic ideals together with new legislations, may present nurses working within acute psychiatric care with conflicting interests and challenges that may cause moral distress.

Our research questions are: May the ideal of reduced use of restrictive, restraining, and coercive treatments within mental health care lead to moral distress? If so, in what way may this lead to moral distress?

## Methods

This paper is part of a larger study on sources, features, and reactions to moral distress in nurses working in acute psychiatric settings [13]. Nurses’ insider perspective on the moral challenges in psychiatric treatment and care and how they cope with this is so far insufficiently studied [20, 21, 23]. Hamric [24] points out that qualitative studies sensitise us to the more complete and nuanced understanding of moral distress and locates moral distress in the specific contexts in which it occurs. A qualitative design was therefore chosen as we wanted to learn about the interviewees’ subjective experiences, attitudes and thoughts [25, 26]. Such designs are well-suited to study this complex moral phenomenon and how moral agents experience moral distress in dynamic contexts [27]. The empirical data collection took place in two different hospitals in the south-eastern part of Norway.

This became a three-part study: Individual interviews were conducted in 2017 shortly before *Law on Patients’ Rights* was implemented. Although not a planned topic, the possible practical consequences of this upcoming law was often discussed by the interviewees. The second set of individual interviews was conducted in 2018, after the implementation of the law. The practical consequences this change in legislature and how the ideal of reduced use of restraints and coercion influenced their practice, became a focal topic.

Three focus group interviews with a total of fourteen participants were organised to delve deeper into these matters. Also during these interview sessions moral distress were highlighted. Each interview lasted about 90 min. We chose this method to learn about the nurses’ experiences working according to the political and therapeutic ideal of reducing the use of restraints and coercion and to utilise the synergy of thoughts and associations that focus group interviews may create [28].

During the individual interviews and the focus group interview sessions follow-up questions and «mirroring» were used to develop, clarify, and verify statements [13]. In total, 30 nurses were interviewed (Table 1), five of them men. For all three sets of interviews a purposive sampling strategy was used to identify potential participants. The heads of the respective acute psychiatric units in each hospital helped communicate the study’s content, purpose, and goal to all members of their nursing staff orally and in writing. Nurses who were interested in participating, gave their names to the unit leader who forwarded a list to the first author. These were invited to participate in the study. The names of the focus group participants were not given beforehand. *Inclusion criteria were:* Registered nurses with varied length of work experience in the field. Most participants had postgraduate qualifications in psychiatric nursing (Table 1)**.**

**Table 1:** Time of interviews. Background and number of nurses interviewed

Years of psychiatric nurse experience	Interviews before introduction of the *Law on Patients’ Rights*	Interviews after introduction of the *Law on Patients’ Rights*		No. of psychiatric nurse specialists among the interviewees
	Individual interviewees (2017)	Individual interviewees (2018)	Focus group interviewees (2019)	
0–10 years	3	5	10	8
11–20 + years	5	3	4	10
	8	8	14	

### Data analysis

The analysis of all the interviews were thematic and hermeneutic in character. Both the transcribed individual interviews and the focus group interviews were analysed according to Braun and Clarke’s [29], thematic analytic approach. These authors [29], p.79 define thematic analysis as “a method for identifying, analysing and reporting patterns (themes) within data”. They propose two possible ways for identifying themes: inductive (bottom-up) based on what is in the data, or a more “top-down” fashion. The latter is analysis-driven, guided by the researcher’s theoretical or analytical interest in the subject area where the researcher “uses the data to explore particular theoretical ideas or bring those to bear on the analysis being conducted” [30], p.178. Both ways to identify themes were utilised as the rationale for the focus group sessions stem from findings in the individual interviews about the use of restraints and coercion and the presupposed (set 1) and effectuated (set 2) new law.

Braun and Clarke [29, 30] present six phases for thematic analysis: 1) The authors familiarise themselves with the interview data. This we did through reading and re-reading the interview texts actively searching for meanings and patterns. During this first analysis reflective thoughts were documented to help find what main themes were identified in the data. Moral distress became one such theme although none of the interviewees used this term.

The interviews were then re-analysed theme by theme. During each re-analysis interesting features were coded (phase 2). We kept revisiting the interviews to make sure our coding was in line with the empirical data. During phases 2 and 3, the collation of potential sub-themes, our thoughts and ideas evolved from engaging with the data.

In phase 4 we reviewed this paper’s main theme together with its sub-themes and collectively discussed whether they reflected the meanings evident in the data as a whole. Thus, phases 4 and 5 were closely related, as we in phase 5 discussed whether the sub-themes we had developed described the theme’s content. 6) The first author wrote a preliminary paper text which then was discussed and developed further collaboratively. Finally, we returned to the transcripts to ensure that our interpretations were supported by the data material.

Analytic credibility was obtained through quotations with the interviewees’ own description of thoughts and experiences [31]. This also strengthened confirmability and trustworthiness as the quotations show that the findings are based on our interviewees’ responses and not on potential bias (ibid.). Trustworthiness is also strengthened through transferability being achieved by presenting thick descriptions to show that the study’s findings can be applicable in similar contexts, circumstances, and situations.

Being four analysts with different professional backgrounds, two with an insider view as psychiatric nurses and two from other fields of expertise, also helped avoid analytic bias. This furthermore helped secure rigor as we initially re-read the interview texts separately, striving for depth of understanding through a circular investigation of the texts [32] and doing our best to “remain open to the meaning of the other person or the text” [32], p.281. This kind of openness is predicated on a willingness to ‘listen’ to the text and to go where the data lead. This, and our collective discussions of our findings added to the depth of our reflection and thus, to the validity of the analysis.

### Ethical considerations

The study was approved by the Norwegian Social Science Data Services. All interviewees were informed orally and in writing that participation was confidential and voluntary and that they were free to withdraw from the project at any time. There was no pressure to accept the invitation to be interviewed and no negative consequences for those who chose not to sign up for the study. All participants signed a consent form. Institutions and interviewees were made anonymous during the transcription process. Transcriptions and recordings are stored according to ethical research guidelines [33]. Recorded interviews will be deleted on conclusion of the project.

## Results

Through the individual interviews it became clear that the interviewees were preoccupied with the practical consequences of changes in the political, legal, and therapeutic ideals for the treatment of psychiatric patients, particularly the expectations concerning reduced use of restraints and coercion. This topic was expanded upon during the focus group sessions.

The interviewees found that the expectation to minimise the use of restraints or coercion came from politicians, the hospitals’ policy, and unit leadership, and from co-workers. Several of the interviewees found that this gradually made them more open to explore other solutions than restraining and coercive measures. Moreover, their threshold for administering physical restraining measures had become higher.

Even so, the interviewees offered rich descriptions of how these ideals and new legal guidelines also could cause moral distress. Four areas regarding this will be discussed: 1) Challenging behaviour and risk of violence; 2) Minimising the use of restraints and coercion created uncertainty; 3) Legal changes may frustrate treatment; and 4) Consequences for the nursing staff.

### Challenging behaviour and risk of violence

An increasing number of patients have serious mental problems due to synthetic drug use. The nurses found that this, in combination with reduced number of beds in psychiatric units, had resulted in the patients currently admitted being more ill than, say, ten years ago. They experienced a greater tendency to physical violence, like scratching, kicking, blows and strangle-holds as well as serious verbal threats.

One of the nurses had had her ribs broken several times when trying to calm patients down. Patients spitting at the nurses and throwing object were not uncommon. Violent episodes had moreover resulted in broken curtains, pictures, and lamps on the wards. This development was described thus: “We endure more challenging situation for longer than we perhaps should sometimes, with unrest, threats and destruction lasting for a long, long time.”

The unpredictability and feelings of constantly «being on tenterhooks» were described as draining. Having potentially very violent patients moving freely among unsuspecting or anxious patients while they were waiting for transfer to a security unit was also seen as problematic. One of the nurses characterised the scenarios that could be played out on the ward as “limitless and surrealistic”.

Sometimes even co-patients were hurt. Patients had been beaten, threatened, and exposed to co-patients who were “in their face”. Once a patient pulled out another patient’s urine catheter. Afterwards the attacker calmly sat down although the injured man was bleeding profusely. Once a young female patient was attacked by a male patient. He forced her onto the floor and held her down. Later this same patient also managed to enter her room. “When people are howling in the corridors and the ward is in a chaos and alarms go off left and right, patients often want us to lock their door, but this we cannot do as we are responsible for them. But I do understand them …” an interviewee said. “Such conditions are unworthy”.

Unfortunate episodes created an apprehensive mood on the ward and were hard on vulnerable patients and made them retire to their rooms. This made it difficult for the nurses to offer the kind of therapeutic and beneficial environment they needed. Several interviewees described feelings of guilt when unable to safeguard patients who were mentally or physically attacked by co-patients. As a consequence, the nurses’ focus tended to shift from treatment and care to risk evaluation and safety.

### Minimising the use of restraints and coercion created uncertainty

In spite of the patient population’s increasingly poor mental state the expectation to avoid restraints and coercive treatment was strong. It was strong “regardless of whether a hospital uses coercion a lot or sparingly. It is to be reduced”. Some held this to be a requirement coming from «the outside», without explaining this statement further. Others perceived the leadership and/or colleagues to be the source. However that may be, a dilemma arose when it came in conflict with safeguarding patients and staff. Many of the interviewees felt obliged to tolerate more challenging – even violent – behaviour then they previously had done. Restraints and coercion were no longer used until the risk of violence was imminent. Even so,

«It is difficult to know when we actually should use coercion. We try and try and try again all the measures at our disposal to avoid tight-holding or belts. When the patient in spite of this goes on and on and on ... You get to a point when this is enough [has to be stopped]. But you don’t want to use coercion. Afterwards one may think what is right? What is wrong?».

Thus, where to draw the line for patients’ behaviour was seen as difficult:

«Where to draw the line? When does it become dangerous? When someone throws something in the wall? You never know if he’ll attack someone else next time. There is a lot of frustration that is not turned against us, but someone who is screaming and yelling and perhaps throws chairs around is rather threatening. These situations affect us ...».

A nurse fresh out of college worried that other staff members’ expectations would make her «go too far» without elaborating this*.* Another found it difficult as «one does not know where one is supposed to draw the line as one handles things differently and have different limits». Sometimes the nurses wondered whether they or the patients were in control. Hindsight sometimes told them that they should have acted earlier to ensure everyone’s safety. Some interviewees said they rarely discussed how much threats and violence they were expected to tolerate and endure.

The interviewees held that they lacked the predictability and clear limits they needed to make patients and staff feel safe and secure. According to them, it was also a problem that the doctors tended to hesitate to prescribe restraining or coercive measures even when patients potentially could be violent. An interviewee said that she experienced “that one tries to avoid give the order, decisions that are seen as violating personal integrity, even when it could be a very helpful”. Some believed the reason for this could be that the doctors were expected to improve the statistics on the use of such measures and were worried about reactions from outside bodies like the Healthcare Complaints Commission if they did. This could lead to challenging and potentially dangerous situations for the nurses: «I have more than once experienced that the doctor on duty has ordered us to release a patient [from restraints] who still is in a drug induced psychosis and where nothing has changed during the last hour … still as aggressive toward us.»

The nurses tended to “go the extra mile and endure a lot, a lot … we also let co-patients endure a lot, really». Thus, the cost of limiting the use of restraints and coercion could limit the quality of patient care through letting innocent patients endure disturbances and violence on the ward, violence that could be aimed at them. This worried many of our interviewees.

The ideal of minimising the use of restraints and coercion seemed also to come from within. There seemed to be a general agreement among the interviewees that less use of such measures strengthened the patients’ dignity. One described the changes in this area of clinical practice as a relief. She regretted that she previously had taken part in restraining and coercive measures such as placing patients in belts over longer periods of time.

Even so, they all had experienced that restraints at times were necessary to stop patients from hurting themselves, other patients, or staff members. When unavoidable, such measures were described as care, although «these days this is a politically incorrect view». The current therapeutic ideal tended toward letting patients «run off steam» to avoid acting out and violence. Patients who were being very loud but not threatening, who talked directly to co-patients and generally occupied a lot of «space» could make co-patients feel insecure. "Whether we should accept such verbal acting out” caused a lot of discussion among the nurses. “The other patients hear this, too … there can be a lot of shouting, and several patients have said that this makes them anxious”. One of the nurses thought that as a patient “I would never have felt safe on a psychiatric ward”. Another held that being an acute psychiatric patient “is very terrible. To be locked in on an acute ward against one’s will and then having such experiences! It weighs very much on my mind afterwards.”

Having to prioritise threatening or very resource-intensive patients, left less time to follow up on other patients. Thus, the nurses found that the aim to reduce the use of restraints or coercion exposed some patients to greater risk of violence.

### Ideals and legal changes may frustrate treatment

According to the interviewees, due to the new legislation’s strict criteria for involuntary hospital admission many patients who previously had been involuntarily admitted were now admitted on a voluntary basis. It was described as sad and frustrating to witness the gradual decline in patients who refused to receive help: “A great quandary which becomes more and more common, really, is concerning patients with mania who now tend to be admitted voluntarily, who perhaps are on the way “up” and very poorly.” This was experienced as a particularly difficult dilemma when it made children suffer because ill parents could not be retained on the ward against their will. “Some family members are desperate and don’t want their loved ones to be discharged, but there is no legal basis anymore on which to hold them the way we would have done a few years ago”. Untreated patients’ mental health may deteriorate to a point where their actions lead to «economic problems, poorer somatic health, and their messing up their lives quite severely before they are [involuntarily] admitted”.

Several interviewees said they often felt personally responsible for motivating patients to stay in hospital and accept treatment. However, when pressed for time their communication tended more towards persuasion or pressure than motivation: “How hard can you motivate someone to stay, voluntarily, without it becoming, how should I put it, a kind of concealed coercion?”.

Freer access to social medias through being allowed to keep their mobile phones on the ward made it possible for psychotic patients to socially disgrace themselves: “Our hands are tied until they have crossed the line, like when the patient has ruined a relationship, sent their employer nude pictures, for instance”. Such actions may be difficult for the family, too, particularly the patients’ children: “One thing is to disgrace oneself, but you have the family setting which may be totally on the breaking point”.

According to the new legislation longer observation time is required before a decision on involuntary administration of medication can be made. This could result in more use of restraints and coercion and longer hospital stays than necessary. One of the nurses said that her greatest moral challenge at work was to witness how obviously psychotic patients had to wait for days without medicines and adequate treatment. She felt that many of her colleagues agreed with her, but she was one of the very few who discussed this with the leadership.

The nurses worried that the legislation and the ideals concerning reduced use of restraints could affect the most vulnerable patients negatively. They wondered whether politicians ever discuss the needs of this particular group of psychiatric patients when they make decisions on restraining or coercive measures in psychiatric care. As one put it:

«They [the patients] are unable to take care of themselves and many have suffered many losses because of their illness; they have lost their house, their family, their jobs, and finally friends and family cannot take it anymore … I find that society does not take responsibility for the individual. [Politicians] hold that the individual may decide for themselves, basing their opinions on their own lives as resourceful people, and I find that I never would have liked to be treated like this. Would you have liked to lose everything, rejected, in the gutter?”

The interviewees missed a greater focus on the quality of treatment, on dignity and on care when restraining or coercive measures are unavoidable. This perspective seemed to be overshadowed by the seemingly sole focus on reduction of the use of such measures.

### Consequences for the nursing staff

Although restraining or coercive measures at times were necessary, using them often left the nurses with a very unpleasant feeling: «In the morning [after having worked the weekend] you sit together with all the doctors and healthcare staff and have to defend the use of restraints. You feel defeated and perhaps a little ashamed for having had to use such measures». This in spite of having followed doctor’s orders and at the time assessed the measures used as unavoidable. Even though they were not directly criticised for having used restraints or coercion, the nurses could experience defeat and shame. While one claimed that he never had had such reactions, others held that such feelings were difficult to put into words and was something they had never reflected upon previously. One said that “it is the borderline between restraints/coercion, voluntariness, and participation which is so very difficult to contend with”.

Criticism of psychiatric care from various media, the descriptions of psychiatric units as torture chambers, and accusations of violation of human rights, were also experienced as difficult. The interviewees described feelings of isolation because society, family and friends were unable to appreciate what it is like to work on an acute psychiatric ward, the intense experiences, the dilemmas, and the fear. Some were upset by the seeming lack of appreciation among the national political leadership of the severity of the patients’ suffering and the massive challenges healthcarers faced on a daily basis on acute psychiatric wards. The nurses claimed that the groups of psychiatric patients that were discussed in various contexts were far less ill and more resourceful than the patients our interviewees cared for. As their patients were unable to take part in discussions in the media they were hardly ever recognised or heard.

Several of the nurses were furthermore upset by the lack of attention paid to violence from patients and how this also made healthcarers suffer. The interviewees expressed discomfort, guilt, bad conscience, and a feeling of inadequacy when unable to protect patients and staff during episodes of mental and/or physical violence. Such episodes «made the chest physically hurt». Overcrowded wards, inadequate resources and economic saving schemes worried the nurses as it threatened to frustrate their ability to maintain good quality care. Several found that a low staffing ratio led to more use of restraints and coercion. “With more staff we could have solved this differently. It is a perpetual problem”. Particularly on evening shifts and during the weekends, there could be low coverage of nurses and an extensive use of unqualified staff.

Time, competency, and experience were seen as decisive for being able to recognise signals in time to deescalate dangerous situations. However, this is «difficult when the unit is full to capacity». The ward’s architecture and size were also seen as a hindrance for less use of restraints or coercion: “As the rooms are small, they tend to be overcrowded”. All this could cause «growing qualms about being part of a system I do not find good enough». Reducing the use of restraining or coercive measures while saving on expenses was characterised as irreconcilable ideals. Some of the interviewees suffered from tension headaches, others found that exhaustion caused them to be irritable at home and in great need of rest and quiet.

## Discussion

Our results indicate that the political and therapeutic ideal of reducing the use of coercive treatment measures in acute psychiatric care may have a twofold effect: While it may ease the experience of moral distress in nurses who perceive such measures as professionally and ethically problematic, the ideal is hard to adhere to in clinical practice and adhering to it may cause moral distress, as well. The discussion will be focused on 1) Moral doubt and dilemmas regarding how much they should endure on their own and their patients’ behalf, 2) Bearing witness and trapped by the policy imperative; more severely ill patients and insufficient resources and 3) Experiences of guilt, shame, and defeat.

### Moral doubt and dilemmas regarding how much they should endure on their own and their patients’ behalf

That the interviewees’ work environment is increasingly marred by fear due to offensive and violent behaviour from patients is a cause for concern. According to various interviews with mental healthcare workers violence and problems concerning patients acting out are escalating [34]. Repeated exposure to aggression puts nurses at risk of vicarious trauma, occupational stress, PTSD-symptoms, guilt, shame, self-blame [35], muscle and skeletal problems [36] and burnout syndrome [37], and may have significant implications for the quality-of-care provided [38]. The combination of a more seriously ill patient population and the non-restraining and non-coercive treatment philosophy makes it difficult for the nurses to know to what extent they should tolerate patients’ disruptive behaviours. Uncertainty regarding where to draw the line probably causes nurses to be more exposed to threats and violence. Thus, the healthcare personnel have become burdened with the ethical dilemma incorporated in the non-restraining ideal: how much disquiet and psychological and physical violence must co-patients and healthcare personnel endure before the use of restraints is justified?

Internal constraints as socialisation to follow orders [27], loyalty [13], or compliance [39] may be among the reasons why our interviewees seemed faithful to new expectations even though this obviously may be costly to both themselves and patient safety.

Our interviewees seemed to see being brave, unafraid and uncowardly as moral qualities [13]. Being responsible for their own safety was never mentioned. Pettersen and Hem [40] point out that “[c]aring is viewed as an unselfish, spontaneous and compassionate act during which the immediate interests and needs of the particular other should take precedence over the interests of the carer” p217). Not showing feelings and coping with all kinds of situations is often seen as part of being a good nurse [41]. Inability to cope may lead to shame and guilt and to denial of symptoms of stress as «no-one wants to be the cripple of the pack» ([42, p.18]). These ethical, professional, and cultural ideals may contribute to it being challenging for nurses to recognise and put their own moral boundaries into words. Maybe we lack an ethical language to express the complex moral challenges within this area? Moreover, the ideal of reduced use of restraints and coercion is such a strong part of current mental health philosophy that problematisation has become politically incorrect, which may silence anyone who would like to discuss it. The interviewees were strongly focused on how these ideals exposed patients to greater risks of abuse, violence, and insecurity.

The desire to avoid restrictive practices and ensure that all other strategies were exhausted sometimes led the nurses to think that they might have prevented injury to others if they had used restraining measures sooner. This seemed to create moral distress. Even so, the ethical, professional, and legal requirements to keep people safe [43] and the obligation to protect the autonomy of each individual patient constitute a complicated ‘moral enterprise’. Studies show that patients expect to be kept safe from harm and acts of aggression to themselves or others [44, 45]. Our findings indicate that the ambition to reduce the use of restraints and coercion at times may overshadow the ethical concern related to the safety of other patients and a therapeutic atmosphere. This moral dilemma seemed particularly present when a patient’s behaviour created an atmosphere of insecurity and uneasiness, but no obvious threat is present. The moral defensibility of intervention seemed clearer in the presence of an obvious high threat of violence.

In her study on patients' expectations and experiences of feeling safe in an acute psychiatric inpatient ward, Stenshouse [45] found that the perception of threat from other patients was a key issue, highlighting the need to consider patient safety as more than physical safety. However, a treatment philosophy that dictates that nurses must tolerate more unrest among patients to avoid restrictive interventions seems to eclipse the psychological safety of co-patients. Our interviewees worried whether they endured too much on behalf of vulnerable patients who depended on being kept safe by the healthcare personnel.

### Bearing witness and trapped by the policy imperative; more severely ill patients and insufficient recourses

If the idealistic focus on reducing the use of restraining or coercive treatment frustrates the nurses’ ability to offer their patients the best possible care, this may create moral distress. Several interviewees talked about the burden of *bearing witness to* insufficient treatment, for instance patients who due to the longer observation time now required went unmedicated for days, who were in danger of serious decline because they refused treatment, who disgraced themselves, without being able to exercise what they saw as their responsibility and professional duty. The concept of bearing witness may be described as “attesting to the veracity or authenticity of something through one's personal presence” [46], p.289. Bearing witness while having one’s hands tied may lead to moral distress.

Although the nurses wished to reduce the use of restraints and coercion, they seemed to feel trapped if their ethical and professional judgement told them that such measures were unavoidable. Moreover, while the wards often were overcrowded with increasingly ill patients, many of whom suffering from synthetic drug use and prone to violence, our interviewees found that they had to cope with reduced resources. This is in line with an Australian study where “the impact on ward safety with increasing acuity of consumers plus the presence of forensic consumers and those affected by methamphetamine was emphasized” [47], p.1511 was severe. Studies also show that mental health nursing in several countries face an acute workforce crisis and reports on poor staffing levels [48–51].

Concurrently with this development the ideal of reducing the use of restraints and coercion is gaining force, an ideal that could only be successfully implemented with increased resources, according to our interviewees. This is supported by other studies e.g [48, 52–54]. In line with our findings, several studies point to an “association between a lower staff-patient ratio (i.e. less staff members for each patient) and an increase in the use of coercive measures” [3], p.452, the importance of knowledge and competence for the reduction of such measures [55] and an association between high ward occupancy/crowding and aggressive incidents [56–58].

Lack of needed resources in clinical practice seems to be an external constraint which frustrate the nurses’ ability to realise the ideal of reduced use of restricting measures. This creates a dissonance between their values and their actions, which may lead to moral distress. According to Shafer-Landau [59], moral standards that are impossible to meet are illegitimate. To expect healthcare personnel to contribute to the reduction of the use of restraints and coercion in settings with limited resources, where they are responsible for the safety of patients and colleagues while they themselves are scared, may perhaps seem to be an immorally high requirement?

### Experiences of guilt, shame, and defeat

The ideal and expectation of reduced use of restraints and coercion seem in our society to create feelings of guilt, defeat, and shame in nurses after having participated in such treatment measures. Our interviewees described these same reactions when unable to protect patients and staff. These feeling are known features of moral distress. Also persuading the patient to stay on the ward can cause guilt and uneasiness. Andersson et al. [60] found that the use of informal coercion may cause guilt, shame, and self-accusations. According to Doedens [3] nurses more often report feelings of failure, guilt and regret associated with restraining and coercive measures. Others feel ”trapped between the policy imperative for the nurses to protect themselves and others, with nurses ultimately ‘being the scapegoats of the system’” [47], p.1518. The media coverage of mental illness and the treatment of psychiatric patients have been influential in shaping the public’s understanding and attitudes towards involuntary treatment, especially among people with no personal experience with mental illness [61]. Muir-Cochrane et al. [47] found that nurses were worried that society in the future will blame them both for using coercion and for the negative consequences of not using coercion.

The feeling of shame is often activated by unrealistic ideals and high expectations we ourselves create or adopt from others [62]. Shame has been found to be more painful than guilt as not only one's behaviour but one's core self is at stake [63]. Shame may also influence our empathic ability [63]. These kinds of emotional reactions tend neither to be clearly linguistically expressed nor be consciously recognised by those who experience them. Bartky [64] highlights the profoundly disempowering drive for secrecy and concealment induced by shame, which undercuts the possibility and solidarity with others, even those who may be struggling in similar ways.

### Strengths and limitations

The majority of the interviewees were psychiatric nurses with many years’ experience from acute psychiatric care. As participation was voluntary, we cannot say whether the views presented in this paper are representative for all nurses in the hospital units in question. Even so, this study captures pivotal ethical concerns among nurses working in acute psychiatric settings and provides new insights into moral challenges experienced when attempting to solve the tension between clinical realities and the society’s ideals, policies, and legislation. Although our study is limited and local, we believe the insights offered are transferable to other acute care mental health nursing contexts and thus may help decrease the current paucity of knowledge within this field.

## Conclusion

Mental health nurses working in acute psychiatric care are involved in a complex interplay between political and professional ideals to reduce the use of restraints and coercion while being responsible for the safety of both patients and staff as well as creating a therapeutic atmosphere. They often face moral challenges when attempting to solve the tension between ideals, policies and legislation concerning the use of restraining and coercive treatment measures on the one hand and clinical realities on the other. Our findings show that systemic factors like legislations and political and societal ideals may strongly influence on the individual healthcare workers’ responsibilities and working conditions. Caught in the middle nurses/healthcare workers may experience moral distress that may lead to physical discomfort, uneasiness and feelings of guilt, shame, and defeat. High expectations concerning the avoidance of using restraints or coercion in treatment and care and vague guidelines on where to draw the line may also create moral doubt and dilemmas. External constraints like inadequate resources may furthermore hinder the healthcare workers/nurses from realising the treatment ideals set before them. The nurses even worry that new legislation and ideals may prevent acutely psychiatric ill and vulnerable patients from receiving the treatment they need. They are also concerned that their ability to create a psychologically and physically safe environment on the ward may be compromised. This may expose patients and healthcare personnel to greater risk of violence.

Although more research is needed within this field, our findings point to the following implications for practice:

- The leadership needs to be willing to listen to – and when possible – act on the nurses’ ideas, concerns, and professional judgement.
- The leadership needs to actively encourage an ethical work climate through creating a safe and non-judgemental space for healthcare personnel to address the moral challenges and moral distress they face at work.
- Equip the wards with the needed number of adequately trained and competent staff to cope with the increasingly ill patient population and thus enable them to realise the treatment ideals set before them
- Develop clearer guidelines for how to cope with aggressive and violent patients, including when and how restraining and coercive treatment measures may be used.
- After having administered restraining or coercive treatment measures invite the patient to a conversation about the incident when the patient is ready for this.

## Data Availability

To fully protect the identity of this study’s participants the raw data are not available as the data are only deidentified, not anonymised.
